# Association between Fellowship Training, Surgical Volume, and Laparoscopic Suturing Techniques among Members of the American Association of Gynecologic Laparoscopists

**DOI:** 10.1155/2016/5459147

**Published:** 2016-01-18

**Authors:** Emad Mikhail, Lauren Scott, Branko Miladinovic, Anthony N. Imudia, Stuart Hart

**Affiliations:** ^1^Department of Obstetrics and Gynecology, University of South Florida, Morsani College of Medicine, Tampa, FL 33606, USA; ^2^Center for Comparative Effectiveness Research and Evidence-Based Medicine, University of South Florida, Morsani College of Medicine, Tampa, FL 33612, USA

## Abstract

*Study Objective*. To compare surgical volume and techniques including laparoscopic suturing among members of the American Association of Gynecologic Laparoscopists (AAGL) according to fellowship training status.* Design*. A web-based survey was designed using Qualtrics and sent to AAGL members.* Results*. Minimally invasive gynecologic surgery (FMIGS) trained surgeons were more likely to perform more than 8 major conventional laparoscopic cases per month (63% versus 38%, *P* < 0.001, OR [95% CI] = 2.78 [1.54–5.06]) and were more likely to perform laparoscopic suturing during these cases (32% versus 16%, *P* < 0.004, OR [95% CI] = 2.44 [1.25–4.71]). The non-fellowship trained (NFT) surgeons in private practice were less likely to perform over 8 conventional laparoscopic cases (34% versus 51%, *P* = 0.03, OR [95% CI] = 0.50 [0.25–0.99]) and laparoscopic suturing during these cases (13% versus 27%, *P* = 0.01, OR [95% CI] = 0.39 [0.17–0.92]) compared to NFT surgeons in academic practice*. Conclusion*. The surgical volume and utilization of laparoscopic suturing of FMIGS trained surgeons are significantly increased compared to NFT surgeons. Academic practice setting had a positive impact on surgical volume of NFT surgeons but not on FMIGS trained surgeons.

## 1. Introduction

The depth and complexity of gynecologic surgery are continually increasing due to surgical innovation and a trend towards minimally invasive surgical approaches. Due to this increasing intricacy of laparoscopic and hysteroscopic procedures and conflicting obligations during residency training, gynecology residents are frequently uncomfortable performing a variety of advanced endoscopic procedures at the completion of their training [1, 2]. In 2001, the American Association of Gynecologic Laparoscopists (AAGL) and the Society of Reproductive Surgeons (SRS) collaborated to establish the first fellowship in minimally invasive gynecologic surgery (FMIGS), which standardized a MIGS curriculum and a research requirement [3]. The goal of the fellowship is to provide a standardized training program for gynecologists who have completed their residency so that they may acquire additional skills in minimally invasive gynecologic surgery and serve as a scholarly and surgical resource for the community in which they practice [4].

It has been shown that operative outcomes are directly related to surgical volumes, yet there has been a continual decline over the last several decades in the annual number of procedures performed by gynecologists [5, 6]. From 1979 to 2006, there was an 81% decline in the number of gynecologic surgeries performed by ACOG fellows, from 132 to 25 gynecologic cases per year [5]. This trend has also impacted the rate of hysterectomies performed, the most common major gynecologic procedure performed on women in the United States. The number of hysterectomies performed annually by practicing gynecologists has decreased from 28 in 1980 to 8.5 in 2010. This decrease has occurred despite the increased number of methods available to perform a hysterectomy, which now includes a robotic and laparoscopic approach [4].

There have not been any previous studies comparing surgical volumes for FMIGS graduates to other gynecologic surgeons. Given this void in knowledge on the impact of a formal fellowship training program on surgical volume and laparoscopic surgical techniques, we sought to compare fellowship training status, surgical volume, and the surgical techniques of laparoscopic suturing among members of the American Association of Gynecologic Laparoscopists (AAGL).

## 2. Material and Methods

The AAGL is the leading association promoting minimally invasive gynecologic surgery among physicians worldwide. Its membership extends to over 110 countries, with over 7000 members [4]. Given the mission of AAGL, their members were thought to be the most appropriate sample for this study.

A survey was developed using Qualtrics (Qualtrics, Provo, UT) [7] web-based survey platform. After obtaining exempt status from University of South Florida Institutional Review Board, the survey was sent to the AAGL scientific review committee for approval. The survey was pilot tested to ensure that it was easy to understand and the questions were relevant and to gauge the time needed to complete the survey. Upon approval, the survey was sent to all AAGL members through the AAGL Digital Member Bulletin. Following the initial email, two additional reminders were sent to all members to enhance the response rate. The survey was divided into different sections. The first section focused on demographics, the location and type of residency, and the number of residents per year. The second section focused on individual training and surgical volume while the last section focused on individual preferences and technique of laparoscopic suturing. Performance of a total laparoscopic hysterectomy (TLH) was used as a minimum reference point to allow study participants to proceed into the survey. As TLH is a commonly performed minimally invasive gynecologic procedure that requires advanced laparoscopic and suturing skills, it was considered a good reference surgery for this study. Only study participants who perform TLH were included in this study. We focused on conventional laparoscopic cases and conventional laparoscopic suturing in this survey.

A total of 521 respondents accessed the survey, and 163 respondents were excluded from the final analysis because they do not perform conventional total laparoscopic hysterectomy procedures (*n* = 100), are currently in training (*n* = 35, 5 residents and 30 fellows), listed multiple responses (*n* = 28), or had missing information in their responses (*n* = 28). The remaining 330 respondents were categorized into one of the following groups: (1) non-fellowship trained (NFT) (*n* = 219), (2) minimally invasive gynecologic surgery (FMIGS) trained (*n* = 74), and (3) other training (OT) (*n* = 37). The respondents from other training group were Gynecologic Oncology (*n* = 13), Female Pelvic Medicine and Reconstructive Surgery (*n* = 10), and Reproductive Endocrinology and Infertility (*n* = 14). Given that the emphasis of the study was on non-fellowship trained and FMIGS surgeons, these two groups were the basis of our comparison for this study. Regarding questions comparing laparoscopic suturing zones, any combination of vertical alignment of the needle driver and the assistant instrument was considered as suturing in the vertical zone, and any combination of horizontal alignment of the needle driver and the assistant instrument was considered as suturing in the horizontal zone, and any combination that uses the suprapubic port site was considered utilization of the suprapubic site for suturing.

All data was analyzed using Stata 13.1 (StataCorp LP, College Station, TX) SVY survey data analysis module. SVY module incorporates survey design features such as probability weights, stratification, and clustering. Results were expressed as mean ± SD or median (range) for continuous variables and as frequencies and percentages for categorical variables. For comparisons between groups, mean difference for continuous data or odds ratios for categorical data were used. A *P* value of < 0.05 is considered statistically significant.

## 3. Results

A total of 330 respondents were included in the final analysis, over 66% (*n* = 219) of these were NFT, and 22% (*n* = 74) were FMIGS trained while the remaining 18% (*n* = 37) were trained in other types of fellowships (Gynecologic Oncology, Reproductive Endocrinology and Infertility and Female Pelvic Medicine and Reconstructive Surgery) (Table 1).

The vast majority of NFT and FMIGS trained surgeons were male (78% versus 55%, resp.), and over 50% of the respondents (NFT and FMIGS) were between the ages of 35 and 55 years. About 68% of NFT and 52% of FMIGS surgeons practice in the US and predominately in private practice (75% versus 55%, resp.). A greater proportion of FMIGS surgeons work in academic practice (37.9%) versus private practice (19.9%), *P* < 0.001, Table 1.

Most of the NFT and FMIGS respondents were trained at a university residency program (54% versus 72%, *P* = 0.011) with 1–5 residents per year (61% versus 45%, *P* = 0.07) and over three-quarters of the respondents attended a laparoscopic suturing course (78% versus 87%, *P* = 0.13), Table 2.

Minimally invasive gynecologic surgery fellowship trained physicians were more likely to perform more than 8 major conventional laparoscopic cases a month (63% versus 38%, *P* < 0.001, OR [95% CI] = 2.78 [1.54–5.06]) and they were more likely to perform laparoscopic suturing during these cases (32% versus 16%, *P* < 0.004, OR [95% CI] = 2.44 [1.25–4.71]), Table 3.

The NFT surgeons in private practice were less likely to perform over 8 conventional laparoscopic cases (34% versus 51%, *P* = 0.03, OR [95% CI] = 0.50 [0.25–0.99]) and laparoscopic suturing in these cases (13% versus 27%, *P* = 0.01, OR [95% CI] = 0.39 [0.17–0.92]) compared with NFT surgeons in academic practice. The surgical volume and proportion of conventional laparoscopic cases in which suturing is performed among FMIGS surgeons in academic and private practices were not significantly different (Table 4).

Specific analysis of surgical techniques for TLH between NFT and FMIGS surgeons revealed overall similarities in the two groups except in the use of a curved laparoscopic needle driver, which was more common in FMIGS trained surgeons (66% versus 52%, *P* = 0.03) compared to NFT surgeons. Additionally, NFT surgeons were more likely to utilize self-righting needle driver (22% versus 7%, *P* = 0.003) and less likely to perform continuous vaginal cuff closure during TLH (52 versus 66%, *P* = 0.03).

Comparison of the different laparoscopic suturing zones between NFT and FMIGS surgeons showed no statistically significant difference. However, the most commonly utilized one was the transverse zone suturing used by 71 (45%) of NFT and 25 (45%) of FMIGS surgeons, followed by vertical zone suturing and then suprapubic port utilization.

## 4. Discussion

The findings from this survey amongst AGGL members reveal that surgical volume and utilization of laparoscopic suturing in FMIGS trained gynecologists are significantly higher than those of NFT gynecologists. Interestingly, regardless of training though, the TLH surgical technique between the two groups (FMIGS and NFT) of gynecologic surgeons was generally similar.

Several studies have found that surgical residents perceive a need for additional training in advanced laparoscopic procedures after residency [8–10]. This is also true in obstetrics and gynecology residency training programs. In a study by Burkett et al., it was found that only 28.6% of US obstetrics and gynecology residency program directors, and 22.2% of graduating residents from these programs, reported graduating residents as “completely prepared” to perform a laparoscopic hysterectomy procedure [2]. A study by Kolkman et al. found that 73% of recent graduates from a residency training program in obstetrics and gynecology felt they were adequately trained in basic laparoscopic skills, but 82% felt they were not adequately trained to perform more advanced laparoscopic procedures. A lack of an adequate case load was listed as a significant reason for these findings [11].

Obtaining adequate surgical training has now become even more challenging with residency training work week restrictions, combined with the declining rate of gynecologic surgical volume including hysterectomy procedures, which may result in gynecologists in practice altering their practice patterns and even referring relatively uncomplicated procedures to gynecologists with higher volumes [12]. This may cause the practicing gynecologist to be at a serious competitive disadvantage if they are not proficient in the growing repertoire of endoscopic surgeries [13]. As many OB/GYNs have busy schedules, moving between the office and taking care of obstetric patients, the ability to learn and integrate advanced laparoscopic procedures successfully into daily practice is a major hurdle in the implementation of laparoscopy not learned during residency training [11].

In 2012, according to the American Council for Graduate Medical Education, the average graduating resident in obstetrics and gynecology performed 38 laparoscopic hysterectomies. Considering that the learning curve for a laparoscopic hysterectomy is between 30 and 80 procedures and a US gynecologist only performs an average of 8.5 hysterectomies per year in practice, it could take several years after residency training before proficiency is fully achieved [5, 14–16]. Additionally, two decades ago the graduating resident in obstetrics and gynecology was only required to learn two routes for hysterectomy, open and vaginal, but today the graduating resident and the practicing gynecologic surgeon are required to master multiple approaches to the same procedure: abdominal, vaginal, laparoscopic, and robotic hysterectomy [17]. The above-mentioned numbers also do not take into account the volume of surgery needed to maintain proficiency since performance can deteriorate over time, including laparoscopic suturing skills if not practiced regularly to maintain those skills. Therefore maintenance training has been proposed to ensure better skill retention [18].

In this study, FMIGS trained surgeons in both an academic and private practice perform more complex cases with higher utilization of laparoscopic suturing. This presents a significant challenge for the low volume gynecologic surgeons as surgical volume is increasingly being used as a component in assessment of individual and hospital surgical quality [19]. Recently, surgeon volume requirements have been incorporated into maintenance of certification for cardiothoracic surgery [20]. It is evident from this study that the surgical volume of FMIGS trained surgeons is significantly more than that of NFT surgeons. The effect of surgical volume on perioperative outcomes and resource utilization has also been demonstrated in the literature. In a study by Wallenstein et al., it was found that women operated on by high volume surgeons (>14.10 operations per year) were 25% less likely to experience a complication, while women undergoing a surgical procedure at a high volume center (>105 laparoscopic hysterectomies per year) were 18% less likely to experience a complication. Procedure costs were also lower for high volume surgeons ($867 lower costs) and when the procedure was performed at a high volume center ($966 lower costs) [6]. There appears to be a dose-response relationship between surgical volume and outcomes more than a strict cutoff level [6, 19, 21].

This study showed that not only does additional postresidency laparoscopic training enhance a gynecologist's surgical volume, but also the type of practice setting plays an important role. We found that NFT gynecologists practicing in an academic setting had an increase in their surgical volume compared to their peers in private practice, a finding that was not found in the FMIGS group since they had a higher surgical volume regardless of their practice. The hybrid obstetrician-gynecologist career can be uniquely vulnerable to available surgical volume, which could ultimately limit major operative cases to select surgeons [19]. On the other hand, gynecologic surgeons who have a high surgical volume are more likely to feel comfortable offering a minimally invasive hysterectomy to their patients [22]. Laparoscopic suturing and knot tying require development of a significant skillset requiring patience and a long learning curve, which can be difficult to achieve despite many advances made in the field [23]. This study shows that postresidency FMIGS training enhances the surgical volume of practicing gynecologist, including those cases requiring laparoscopic suturing. While this study emphasizes the importance of MIGS fellowship training in our specialty, survey studies such as this have some inherent limitations such as recall, response, and researcher biases.

Certain limitations were obvious in this study, including low sample size due to low response rate (10%), despite sending 2 reminder emails to encourage members to respond.

## 5. Conclusion

In summary, this study analyzed surgical volume between FMIGS and NFT surgeons and showed that surgeon volume among FMIGS trained gynecologists is higher and they are more likely to perform advanced laparoscopic cases involving laparoscopic suturing. Further prospective studies evaluating the benefits of postresidency advanced laparoscopic training on enhancement of perioperative outcomes are needed.

## Figures and Tables

**Table 1:** Demographics of study participants.

	Non-fellowship trained	FMIGS	Gynecologic Oncology	FMPRS	REI	*P* value
	*n* = 219	*n* = 74	*n* = 13	*n* = 10	*n* = 14	
Male	171 (78%)	41 (55%)	10 (77%)	5 (56%)	14 (100%)	<0.001
Female	48 (22%)	33 (45%)	3 (23%)	4 (44%)	0 (0%)	
Age						
25–34	9 (4%)	11 (15%)	0 (0%)	0 (0%)	0 (0%)	<0.001
35–44	49 (22%)	37 (50%)	3 (13%)	4 (40%)	3 (21%)	
45–55	65 (30%)	19 (26%)	6 (46%)	3 (30%)	2 (14%)	
>55	96 (44%)	7 (10%)	4 (31%)	3 (30%)	9 (65%)	
Geographical location						
US	146 (68%)	38 (52%)	6 (50%)	6 (60%)	7 (54%)	0.10
International	69 (32%)	35 (48%)	6 (50%)	4 (40%)	6 (46%)	
Practice type						
Academic practice	54 (25%)	33 (45%)	9 (69%)	5 (50%)	5 (36%)	<0.001
Private practice	165 (75%)	41 (55%)	4 (31%)	5 (50%)	9 (64%)	

**Table 2:** Training background for study participants.

	Non-fellowship trained (219)	FMIGS trained (74)	*P* value
Residency training			
University	117 (54%)	53 (72%)	0.011
Community-university affiliated	58 (27%)	16 (22%)	
Community	41 (19%)	5 (6%)	
Residency program size			
1–5	130 (61%)	33 (45%)	0.07
6–10	59 (27%)	28 (38%)	
>10	25 (12%)	12 (7%)	
Attended laparoscopic suturing training courses			
Yes	169 (78%)	64 (87%)	0.13
No	49 (22%)	10 (13%)	

**Table 3:** Case volume of study participants.

	Non-fellowship trained	FMIGS trained	*P* value	OR (95% CI)
Major gynecologic conventional laparoscopic surgical cases/month				
>8	81 (38%)	45 (63%)	<0.001	2.78 (1.54–5.06)
Monthly cases with conventional laparoscopic suturing				
>8	34 (16%)	23 (32%)	0.004	2.44 (1.25–4.71)

**Table 4:** Case volume of non-fellowship trained participants according to practice setting.

	Non-fellowship trained		*P* value	OR (95% CI)
	Academic practice	Private practice		
Major gynecologic conventional laparoscopic surgical cases/month				
>8	26 (51%)	55 (34%)	0.03	0.50 (0.25, 0.99)
Monthly cases with conventional laparoscopic suturing				
>8	14 (27%)	20 (13%)	0.01	0.39 (0.17, 0.92)

	FMIGS trained		*P* value	OR (95% CI)
	Academic practice	Private practice		

Major gynecologic conventional laparoscopic surgical cases/month				
>8	23 (72%)	22 (56%)	0.18	0.51 (0.16, 1.52)
Monthly cases with conventional laparoscopic suturing				
>8	8 (24%)	15 (38%)	0.2	1.95 (0.63, 6.31)
